# AN INTERNATIONAL COMPARISON AND CHINA’S REGIONAL DEVELOPMENT: EVALUATION OF QINGDAO’S LONG-TERM CARE POLICY

**DOI:** 10.1093/geroni/igae098.3790

**Published:** 2024-12-31

**Authors:** Huiru Song, Hong Mi

**Affiliations:** King’s College London, Shandong, China (People’s Republic); Zhejiang University, Hangzhou, Zhejiang, China (People’s Republic)

## Abstract

Qingdao is the first city in China to fully implement long-term care insurance, and its system has important reference significance for the healthy and equity development of the elderly population in Shandong Province. The policy divides long-term care insurance benefits into medical services and nursing services, with the medical service fee being 3,500 yuan per year for employees and 3,000 yuan per year for residents, and the nursing service fee being 18,000 yuan per year for employees and 5,000 yuan per year for residents. According to forecasts, the GDP of Shandong Province is expected to reach 14,145.63 billion yuan in 2035. Therefore, according to the ratio of one employee to two permanent residents, under the fixed disability rate program, the province’s disabled elderly population is 1,554,800, including 513,100 employees and 1,041,700 permanent residents, and under the variable disability rate program, the province’s disabled elderly population is 1,728,700, including 570,500 employees and 1,158,200 permanent residents. At the same time, under the fixed disability rate plan, the long-term care insurance expenditure in Shandong Province in 2035 will be 8826,153,360 yuan, accounting for 6.23/10,000 of GDP. Under the variable disability rate scenario, long-term care insurance expenditure is 981,3526,560 yuan, accounting for 6.937 per 10,000 of GDP, which is much lower than that of OECD countries. In the future, Shandong Province should implement precise positioning based on Qingdao’s policies and combine the regional characteristics of each city to promote long-term care insurance from wide coverage to full coverage.

